# Economic contribution and attitude towards alien freshwater ornamental fishes of pet store owners in Klang Valley, Malaysia

**DOI:** 10.7717/peerj.10643

**Published:** 2021-01-13

**Authors:** Abdulwakil Olawale Saba, Ahmad Ismail, Syaizwan Zahmir Zulkifli, Shamarina Shohaimi, Mohammad Noor Amal Azmai

**Affiliations:** 1 Department of Biology, Faculty of Science, Universiti Putra Malaysia, UPM Serdang, Selangor, Malaysia; 2 School of Agriculture, Lagos State University, Epe, Lagos, Nigeria

**Keywords:** Alien species, Ornamental fish, Livelihood opportunities, Socioeconomic, Questionnaire survey

## Abstract

Malaysia is one of the top ten countries in the world that produce freshwater ornamental fishes. This industry can offer better livelihood opportunities to many poor households. However, most of the produced ornamental fishes are alien to Malaysia. In this study, we explore the contribution of alien freshwater fishes to the income of ornamental fish store owners and their attitude towards alien freshwater fishes within Klang Valley, Malaysia. Using a structured questionnaire, we surveyed 70 pet stores out of which 54 (81.42%) store owners responded. Most of the pet store owners were male (72%), Chinese (83%), and the highest educational level was at the secondary level (79%). Most of the pet store owners reported a monthly income of RM 2001–RM 5000 (78%) and were married (73%). Using Chi-square (χ^2^) test, significant relationships (*p* < 0.05) existed between the attitude of store owners towards alien ornamental fish species versus educational level (χ^2^ = 16.424, *p* = 0.007) and contribution of alien ornamental fishes to the pet store owners’ income (χ^2^ = 27.266, *p* = 0.003). Fish sales as the main income source also related significantly with the impact of fish selling business on income level (χ^2^ = 10.448, *p* = 0.007). This study showed that the ornamental fish sales contributed over half of the income (51–100%) from the businesses of store owners. Almost half of the respondents (42%) reported that alien ornamental fish was the highest contributor to their income from the ornamental fish sale. While the mismanagement of alien ornamental fishes could give various negative ecological impacts, the socio-economic benefits of these fishes cannot be denied.

## Introduction

Ornamental fishes are frequently referred to as “living jewels” due to their beautiful colours, varying shapes, entertaining behaviours, and the capability of living in confined spaces (Ng, 2016; Lokman et al., 2019). Moreover, these fishes can offer better livelihood opportunities to many low-income families around the world (Cagauan, 2007). The ornamental fish industry makes a vital contribution to international trade in many developing countries where ornamental fishes are produced. Globally, about 125 countries are involved in the ornamental fish trade, and this is worth about USD 15–30 billion annually (Evers, Pinnegar & Taylor, 2019). Freshwater fishes that occupy 90% of this total are mostly sourced from breeding facilities in developing countries not only in Asia or South America, but also in Israel, USA, and Europe (Raghavan et al., 2013; Evers, Pinnegar & Taylor, 2019). Moreover, 90% of the freshwater fishes in the ornamental trade are bred in captivity with the remaining 10% being caught from the wild (Raghavan et al., 2013).

The global ornamental fish market is dominated by 30 freshwater fish species, and these include various alien fish species for many countries (Dey, 2016; Evers, Pinnegar & Taylor, 2019). Since the 1970s, there has been a yearly growth of 14% in the popularity of fish keeping with over 5,300 species of freshwater fish in the annual global trade (Collins et al., 2012; Maceda-Veiga et al., 2016). At a value of USD 197.7 million, Asian countries accounted for 57% of the trade in the year 2014 (Evers, Pinnegar & Taylor, 2019), and this may be due to all major ornamental fish breeding activities were in this region, supported with low mortality and affordable prices (Monticini, 2010).

Malaysia is one of the top ten countries in the world that produces freshwater ornamental fishes (Evers, Pinnegar & Taylor, 2019; Lokman et al., 2019). Besides, the collection, breeding and marketing of ornamental fishes is a sizable industry that generates foreign exchange and creates jobs for the locals. Nevertheless, ecological sustainability and the economic viability of the industry call for concern (Ng, 2016; Lokman et al., 2019). Moreover, there is existing evidence that the ornamental fish trade is an important route for alien fish introduction and translocation (Gertzen, Familiar & Leung, 2008; Chang et al., 2009; Chan et al., 2019).

However, there appears to be a general lack of information on the contribution of the alien ornamental fish industry to livelihoods in Malaysia. To assist policymakers towards achieving sustainability which consists of the environmental, social, and economic components, it is necessary also to assess the socioeconomic dimensions of the alien ornamental fish species in the country (Saba et al., 2020b). Thus, the objectives of this study are: (1) to determine the relationships between variables measuring socioeconomic contribution and respondents’ attitudes towards alien freshwater ornamental fishes; (2) to compare the attitudes of groups having different demographic characteristics towards alien freshwater ornamental fishes; and (3) to evaluate the contribution of alien freshwater ornamental fish sales to the businesses of pet store owners within Klang Valley, Malaysia.

## Materials and Methods

### Study area

Klang Valley which covers seven major areas such as the Federal Territory of Kuala Lumpur, Gombak, Hulu Langat, Kuala Langat, Sepang, Klang and Petaling is situated at the centre of the west coast of Peninsular Malaysia and covers approximately 2,832 km^2^. It cuts across important cities such as Ampang Jaya, Kuala Lumpur, Banting, Sepang, Klang, Petaling Jaya, Shah Alam and Subang Jaya (Rashid & Ishak, 2009). This area was selected as a result of being urbanized and with a large population of over 4 million people which represents about 16% of the entire country’s population (Naji et al., 2014). It also houses many freshwater ornamental fish stores. This study primarily surveyed all identified and accessible ornamental fish pet stores within Klang Valley and took place throughout the year 2019.

### Questionnaire development and scope

A questionnaire with two sections about demographics (six questions) and socioeconomic aspects of alien freshwater ornamental fish species trade in Klang Valley (15 questions) was developed, all resulting in 21 questions (Supplemental File 1). Questions included dichotomous (yes/no), open-ended (with undefined answering categories), Likert-type (from 1 = ‘‘strongly disagree’’ to 4 = ‘‘strongly agree’’) and multiple-choice responses. To double-check the contribution of ornamental fish to the income of store owners, the same question addressing this was asked in two different ways; one as a dichotomous question and the other as a Likert-type question. The Malay language version of the questionnaire was also prepared through a forward and backward translation of the English language questionnaire.

In the questionnaire, six questions gathered responses on the sociodemographic characteristics of the respondents, including their gender, age group, ethnicity, education, monthly income, and marital status. There were three Likert-type statements with two questions related to the economic contribution of alien ornamental freshwater fish and one related to the attitude of respondents concerning the alien ornamental freshwater fish trade and culture in Malaysia. Two multiple choice-questions were related to information about the fish business, and the number of years respondents had spent in the business, respectively. Three dichotomous questions addressed the sale of non-fish aquarium accessories, fish sales as the main income source, and fish breeding activity. Finally, seven open-ended questions addressed income information, most frequently purchased fish species, percentage of income from the fish selling business, number of fish species available for sale, number of fish sold daily, and amount of money made daily from the fish sale, respectively.

Face and content validation were assessed by three experts in the area of fish ecology. The questionnaire was revised several times based on recommendations until the final version was ready to be used.

### Data collection

Data were collected from the ornamental fish store owners within Klang Valley, Malaysia. Since there was no official sampling frame to determine the total number of stores within the sampling area, this study relied on data from the Department of Fisheries, Ministry of Agriculture and Food Industries, Malaysia and Google maps to collate and locate all available pet stores in the area. At the end of the survey, 54 (81.42%) out of 70 ornamental fish pet stores agreed to respond to the questionnaire. In each pet store, the questionnaire was administered face to face to the owner or a representative of the owner. The list of alien and native ornamental freshwater fish species recorded from pet stores within the sampling area as listed by Saba et al. (2020a).

### Data analysis

Descriptive statistics were carried out using Microsoft Office Excel (Version 2016, Microsoft Corp., Berkshire, UK), while inferential statistical analyses including Spearman’s rank correlation and Chi-square (χ^2^) test of association were done using Statistical Package for Social Sciences (SPSS) (version 22; Armonk, NY, USA). Spearman’s rank correlation was used to identify relationships between the socioeconomic characteristics of store owners and their businesses.

After cleaning, coding, and describing the data using Microsoft Office Excel, Spearman’s rank correlation identified variables that had significant correlation (*p* < 0.05). Furthermore, the influence of demographic characteristics on the attitude of store owners was tested by subjecting data to the Chi-square (χ^2^) test of association (*p* < 0.05). The same test was also used to identify associations between store owners’ attitudes to alien fish in Malaysia with other socioeconomic characteristics. In the same vein, a Chi-square test was conducted to identify possible associations between the income level of store owners with other socioeconomic characteristics.

## Results

### Sociodemographic profiles and response summary

**Figure 1:**
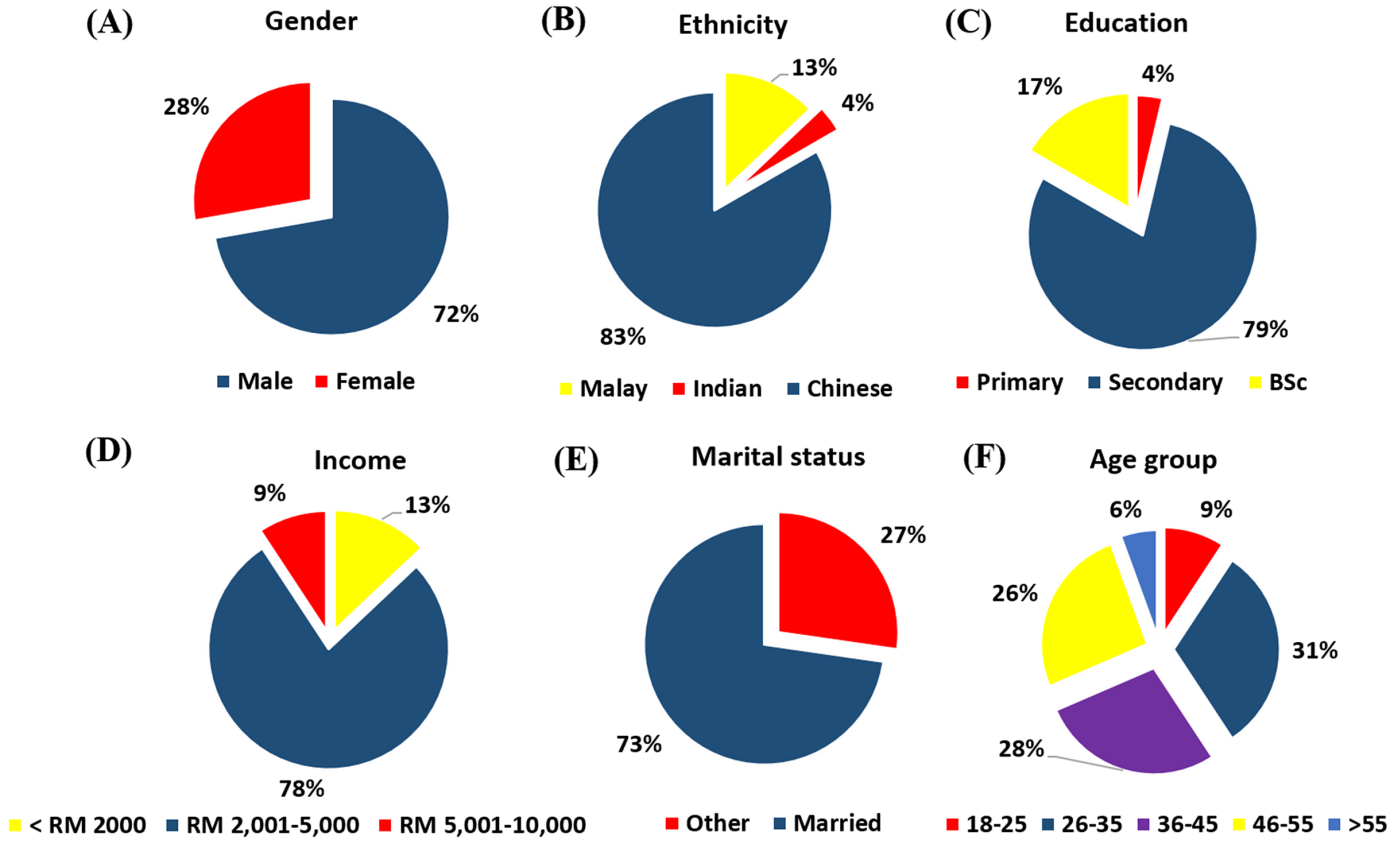
Sociodemographic characteristics of ornamental freshwater fish pet store owners within Klang Valley, Malaysia. (A) Gender, (B) ethnicity, (C) education, (D) income level, (E) marital status and (F) age group.

**Figure 2:**
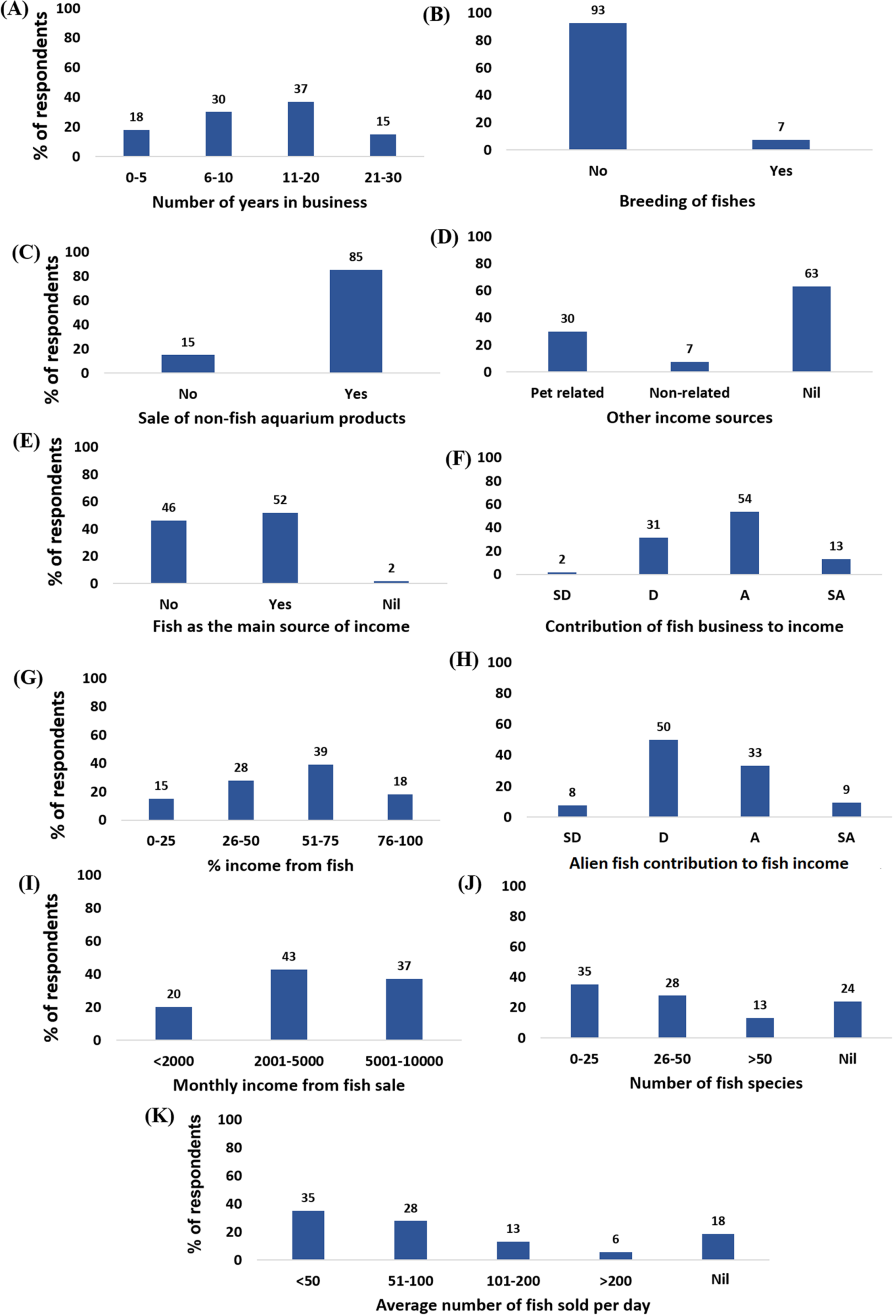
Summary of responses from ornamental fish store owners in Klang Valley, Malaysia. (A) The number of years in business, (B) breeding of fishes, (C) sale of non-fish aquarium products, (D) other sources of income, (E) fish sales as the main source of income, (F) impacts of fish business on income level, (G) percentage income from fish selling business, (H) alien fish contribution to fish income, (I) monthly income from fish sale, (J) number of fish species available, and (K) average number of fish sold/day. SD, strongly disagree; D, disagree; A, agree; SA, strongly agree. Nil refers to the percentages of respondents who gave no response to the particular questions.

**Figure 3:**
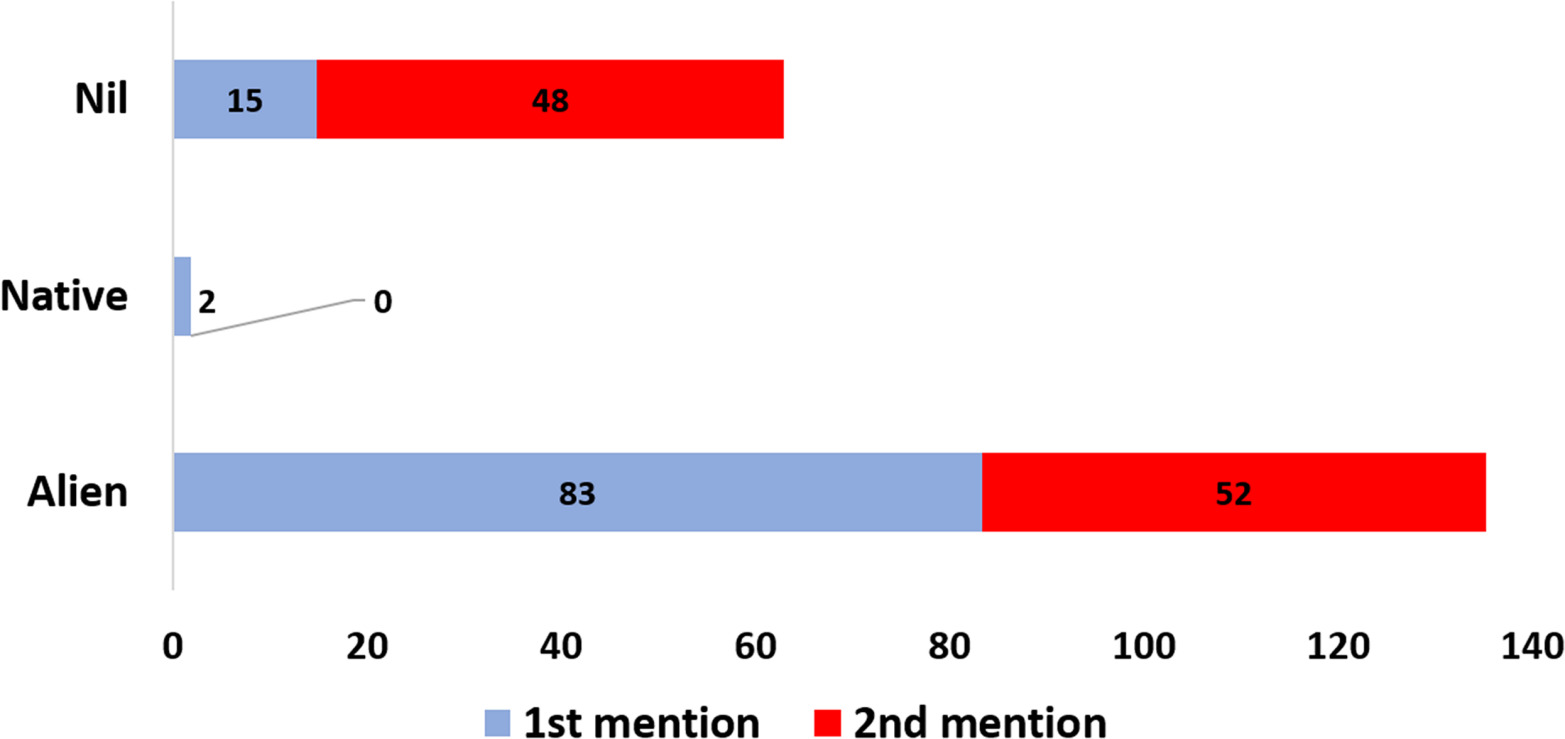
Percentages of the most purchased fish types by origin (native and alien) at first and second mentions by the ornamental freshwater fish store owners in Klang Valley, Malaysia. Nil refers to the percentage of respondents who gave no response to the question about the mostly purchased fish types by origin.

Most of the pet store owners were male (72%), Chinese (83%), and educated at the secondary level (79%). Also, most of them are married (73%), obtained a monthly income between RM 2,001 and RM 5,000 (78%), and fall between the age of 26–35 (31%) (Fig. 1). A summary of responses to questionnaire items by categories of the ornamental fish store owners is presented in Fig. 2. Concerning the percentages of the most purchased fishes by origin (native or alien) at first and second mentions by the respondents, alien freshwater fishes occurred most frequently in both cases at 83% and 52%, respectively (Fig. 3).

### Correlational analysis

Out of the 11 significant relationships (*p* < 0.05) between pairs of items measuring the economic contribution of alien fishes and store owners’ attitudes towards alien fishes in Malaysia, eight relationships showed moderate (*r* = 0.300–0.700) with positive correlations. However, out of the other significant relationships that were weakly correlated, only the number of available alien fishes correlated negatively with the number of years in business (*r* = −0.300; *p* = 0.040) (Table 1).

**Table 1:** Relationships between the pairs of items measuring socioeconomic factors and attitude of ornamental freshwater fish store owners in Klang Valley, Malaysia.

		Incm_lev	Yrs_bus	main_so_incm	Contr_ali	Incmlev_fis	%Incm_fis	Num_avail	Num_sold	Incme_day	Atti_ali_ fish
Incm_lev	*r*	1.000	0.051	−0.074	0.191	−0.148	−0.032	0.268	0.277	0.125	0.237
	*p*		0.715	0.597	0.167	0.284	0.817	0.068	0.069	0.474	0.084
Yrs_ bus	*r*		1.000	−0.019	−0.125	−0.012	**0.289**	**−0.300**	0.097	−0.221	−0.166
	*p*			0.891	0.368	0.933	**0.034**	**0.040**	0.533	0.203	0.231
main_so_incm	*r*			1.000	**0.312**	**0.558**	**0.311**	0.152	0.064	−0.016	0.094
	*p*				**0.023**	**0.000**	**0.023**	0.313	0.682	0.927	0.502
Contr_ ali	*r*				1.000	**0.287**	0.261	0.204	0.170	0.131	**0.463**
	*p*					**0.036**	0.056	0.170	0.271	0.452	**0.000**
Incmlev_fis	*r*					1.000	0.092	0.168	0.015	0.104	0.080
	*p*						0.509	0.260	0.921	0.551	0.564
%Incm_fis	*r*						1.000	0.052	0.031	−0.113	0.202
	*p*							0.726	0.841	0.518	0.142
Num_avail	*r*							1.000	**0.464**	**0.607**	0.138
	*p*								**0.002**	**0.000**	0.354
Num_sold	*r*								1.000	**0.541**	−0.007
	*p*									**0.001**	0.962
Incme_ day	*r*									1.000	**0.340**
	*p*										**0.046**
Atti_ali_ fish	*r*										1.000
	*p*										

**Note:**

Incm_lev = Monthly income level; Yrs_ bus = Number of years in business; main_so_incm = fish as main source of income; Contr_ ali = contribution of alien fish to fish income; Incmlev_fis = contribution of fish business to income level; %Incm_fis = percentage income from fish; Num_avail = number of species available for sale; Num_sold = average number of fish pieces sold/day; Incme_ day = average daily income; Atti_ali_ fish = attitudes with regards to alien fishes. Numbers in bold indicate correlation with statistically significant values at *p* < 0.05.

### Attitude regarding alien fish species

Except for the educational level where most of those in the secondary (79%) category agreed and strongly agreed as opposed to 55% of those in the bachelor’s category that agreed, with a statistically significant difference (χ^2^ = 16.424, *p* = 0.007), all other demographic characteristics such as gender, age and ethnicity showed no significant differences (*p* > 0.05) among the demographic categories (Table 2).

**Table 2:** Chi-square (χ^2^) comparisons of attitude regarding alien freshwater ornamental fishes versus sociodemographic characteristics (gender, age, ethnicity, education, and monthly income) of aquarium store owners in Klang Valley, Malaysia.

Gender	SD	D	A	SA	df	χ^2^	*p*-Value
Total	4 (7.4)	10 (18.5)	32 (59.3)	8 (14.8)	3	0.540	0.956
Male	3 (7.7)	8 (20.5)	22 (56.4)	6 (15.4)			
Female	1 (6.7)	2 (13.3)	10 (66.7)	2 (13.3)			
Age (year)							
Total	4 (7.4)	10 (18.5)	32 (59.3)	8 (14.8)	12	13.229	0.524
18–25	0 (0.0)	0 (0.0)	5 (100.0)	0 (0.0)			
26–35	3 (17.6)	4 (23.5)	8 (47.1)	2 (11.8)			
36–45	0 (0.0)	2 (13.3)	8 (53.3)	5 (33.3)			
46–55	1 (7.1)	3 (21.4)	9 (64.3)	1 (7.1)			
>55	0 (0.0)	1 (33.3)	2 (66.7)	0 (0.0)			
Ethnicity							
Total	4 (7.4)	10 (18.5)	32 (59.3)	8 (14.8)	6	6.602	0.554
Malay	2 (28.6)	1 (14.3)	3 (42.9)	1 (14.3)			
India	0 (0.0)	0 (0.0)	2 (100.0)	0 (0.0)			
Chinese	2 (4.4)	9 (20.0)	27 (60.0)	7 (15.6)			
Education							
Total	4 (7.4)	10 (18.5)	32 (59.3)	8 (14.8)	6	16.424	**0.007**
Primary	0 (0.0)	1 (50.0)	1 (50.0)	0 (0.0)			
Secondary	1 (2.3)	8 (18.6)	29 (67.4)	5 (11.6)			
Bachelors	3 (33.3)	1 (11.1)	2 (22.2)	3 (33.3)			
Monthly income (MYR)							
Total	4 (7.4)	10 (18.5)	32 (59.3)	8 (14.8)	6	4.841	0.578
<2,000	1 (14.3)	2 (28.6)	4 (57.1)	0 (0.0)			
2,000–5,000	3 (7.1)	7 (16.7)	26 (61.9)	6 (14.3)			
5,001–10,000	0 (0.0)	1 (20.0)	2 (40.0)	2 (40.0)			

**Note:**

SD, strongly disagree; D, disagree; A, agree; SA, strongly agree. Values outside and within brackets indicate counts and percentages, respectively. Numbers in bold indicate significant differences at *p* < 0.05. USD 1 = MYR 4.16.

Concerning the comparison of store owners’ attitude towards alien fish species with the number of years in business, fish sale as the main source of income, fish mostly purchased by origin (native or alien), contribution of fish business to income level, percentage of income from freshwater ornamental fish sales, and the contribution of alien freshwater ornamental fishes to income from fish sales, significant differences (*p* < 0.05) were observed only in the numbers of years in business (χ^2^ = 20.372, *p* = 0.009), and contribution of alien freshwater ornamental fish to fish income (χ^2^ = 27.266, *p* = 0.003) (Table 3).

**Table 3:** Chi-square (χ^2^) test of association between attitude towards alien freshwater ornamental fish species versus other socioeconomic characteristics of aquarium store owners in Klang Valley, Malaysia.

Years in business	SD	D	A	SA	df	χ^2^	*p*-Value
Total	4 (7.4)	10 (18.5)	32 (59.3)	8 (14.8)	9	20.372	**0.009**
0–5	3 (30.0)	0 (0.0)	5 (50.0)	2 (20.0)			
6–10	0 (0.0)	2 (12.5)	9 (56.3)	5 (31.3)			
11–20	0 (0.0)	6 (30.0)	14 (70.0)	0 (0.0)			
21–30	1 (12.5)	2 (25.0)	4 (50.0)	1 (12.5)			
Fish as the main income source							
Total	4 (7.5)	9 (17.0)	32 (60.4)	8 (15.1)	3	2.073	0.135
No	2 (8.0)	4 (16.0)	17 (68.0)	2 (8.0)			
Yes	2 (7.1)	5 (17.9)	15 (53.6)	6 (21.4)			
Fish most purchased							
Total	4 (8.7)	9 (19.6)	28 (60.9)	5 (10.9)	3	5.39	0.148
Alien	4 (9.1)	8 (18.2)	28 (63.6)	4 (9.1)			
Native	0 (0.0)	1 (50.0)	2 (40.0)	1 (50.0)			
Fish contribution to income level (MYR)							
Total	4 (7.4)	10 (18.5)	32 (59.3)	8 (14.8)	6	4.841	0.096
<2,000	1 (14.3)	2 (28.6)	4 (57.1)	0 (0.0)			
2,000–5,000	3 (7.1)	7 (16.7)	26 (61.9)	6 (14.3)			
5,001–10,000	0 (0.0)	1 (20.0)	2 (40.0)	2 (40.0)			
Percentage of income from fish							
Total	4 (7.4)	10 (18.5)	32 (59.3)	8 (14.8)	9	9.824	0.346
0–25	2 (20.0)	1 (10.0)	6 (60.0)	1 (10.0)			
26–50	1 (7.1)	4 (28.6)	8 (57.1)	1 (7.1)			
51–75	0 (0.0)	4 (18.2)	15 (68.2)	3 (13.6)			
76–100	1 (12.5)	1 (12.5)	3 (37.5)	3 (37.5)			
Alien fish contribution							
Total	4 (7.4)	10 (18.5)	32 (59.3)	8 (14.8)	9	27.266	**0.003**
SD	1 (25.0)	0 (0.0)	3 (75.0)	0 (0.0)			
D	2 (7.4)	8 (29.6)	17 (63.0)	0 (0.0)			
A	1 (5.6)	2 (11.1)	11 (61.1)	4 (22.2)			
SA	0 (0.0)	0 (0.0)	1 (20.0)	4 (80.0)			

**Note:**

SD, strongly disagree; D, disagree; A, agree; SA, strongly agree. Values outside and within brackets indicate counts and percentages, respectively. Numbers in bold indicate significant differences at *p* < 0.05. USD 1 = MYR 4.16.

### Chi-square comparison of income versus other socioeconomic characteristics

Regarding the comparison of alien freshwater ornamental fish contribution to income from fish sale with the number of years in business, monthly income, fish sales as the main source of income, fish mostly purchased by origin (native or alien), the impact of fish business on income level, percentage of income from fish sales, significant differences were observed only for fish sales as the main income source (χ^2^ = 10.448, *p* = 0.007) and impact of fish business on income level (χ^2^ = 22.378, *p* = 0.004) (Table 4).

**Table 4:** Chi-square (χ^2^) test of association between alien freshwater ornamental fish income contribution versus other socioeconomic characteristics of aquarium store owners in Klang Valley, Malaysia.

Years in business	SD	D	A	SA	df	χ^2^	*p*-Value
Total	4 (7.4)	27 (50.0)	18 (33.3)	5 (9.3)	9	14.782	0.063
0–5	2 (20.0)	3 (30.0)	4 (40.0)	1 (10.0)			
6–10	2 (12.5)	4 (25.0)	7 (43.8)	3 (18.8)			
11–20	0 (0.0)	15 (75.0)	4 (20.0)	1 (5.0)			
21–30	0 (0.0)	5 (62.5)	3 (37.5)	0 (0.0)			
Monthly income (MYR)							
Total	4 (7.4)	27 (50.0)	18 (33.3)	5 (9.3)	6	9.286	0.102
<2,000	2 (28.6)	3 (42.9)	2 (28.6)	0 (0.0)			
2,000–5,000	1 (2.4)	23 (54.8)	14 (33.3)	4 (9.5)			
5,001–10,000	1 (20.0)	1 (20.0)	2 (40.0)	1 (20.0)			
Contribution of fish to income							
Total	4 (7.5)	26 (49.1)	9 (36.0)	18 (34.0)	3	10.448	**0.007**
No	3 (12.0)	16 (64.0)	3 (12.0)	3 (12.0)			
Yes	1 (3.6)	10 (35.7)	15 (53.6)	2 (7.1)			
Income level result from fish business							
Total	4 (7.4)	27 (50.0)	18 (33.3)	5 (9.3)	9	22.378	**0.004**
SD	0 (0.0)	0 (0.0)	0 (0.0)	1 (100.0)			
D	2 (11.8)	13 (76.5)	1 (5.9)	1 (5.9)			
A	1 (3.4)	13 (44.8)	13 (44.8)	2 (6.9)			
SA	1 (14.3)	1 (14.3)	4 (57.1)	1 (14.3)			
Fish most purchased							
Total	3 (6.5)	25 (54.3)	15 (32.6)	3 (6.5)	3	4.321	0.348
Alien	3 (6.8)	25 (56.8)	15 (29.5)	3 (6.8)			
Native	0 (0.0)	0 (0.0)	2 (100.0)	0 (0.0)			
Percentage of income from fish							
Total	4 (7.4)	27 (50.0)	18 (33.3)	5 (9.3)	9	9.046	0.352
0–25	2 (20.0)	4 (40.0)	3 (30.0)	1 (10.0)			
26–50	2 (14.3)	9 (64.3)	2 (14.3)	1 (7.1)			
51–75	0 (0.0)	11 (50.0)	9 (40.9)	2 (9.1)			
76–100	0 (0.0)	3 (37.5)	4 (50.0)	1 (12.5)			

**Note:**

SD, strongly disagree; D, disagree; A, agree; SA, strongly agree. Values outside and within brackets indicate counts and percentages, respectively. Numbers in bold indicate significant differences at *p* < 0.05. USD 1 = MYR 4.16.

## Discussion

This study explores, correlates, and compares the aspects of socioeconomic characteristics of ornamental pet store owners and their businesses, and their attitudes towards alien freshwater ornamental fishes in Malaysia. The fact that most of the store owners are Chinese corroborates their well-known enterprising nature (Keong, 2012). In Malaysia, they have demonstrated the impression of possessing a natural flair for business, particularly the ornamental fish breeding, exportation, and marketing (Keong, 2012). In this study, for most aquarium store owners (58%) in Klang Valley, fish sales contribute over half of the income (51–100%) from their business, and alien freshwater ornamental fishes contribute the most to the income from the ornamental fish sale for about 42% of the respondents. However, since our aim was not to compare the pricing of alien versus native fishes, data on the comparative costing was not recorded in this study.

Direct relationships existed between the store owners’ socioeconomic characteristics with the sales of alien freshwater ornamental fishes. For example, the main source of income was positively correlated with the contribution of alien freshwater ornamental fish to income generation. This means that income from alien freshwater ornamental fish sales increases as the income from general fish sales by the store owners increased. The high percentage of alien fish mentioned as the most purchased fish, compared to native fish mentions by the store owners confirmed their contribution to the income from alien ornamental fish sales. This is in line with the fact that most of the fish species in Malaysia’s ornamental fish business are alien (about 74%) and in the year 2018 alone, the ornamental fish produced in Malaysia was valued at over RM 350 million (Kechik, 1995; DOF, 2007, 2018).

In Malaysia, by the year 2006, there were about 500 aquarium stores in the country with about 550 varieties (native and alien) of ornamental fishes comprising more than 250 species being bred. This fact supports the establishment and acceptability of the retail ornamental fish business and its potential to contribute meaningfully to the livelihoods of Malaysia (Keong, 2012). Despite that, the acceptability of the aquarium hobby and possible dumping of alien fish species into local waters may negatively impact freshwater biodiversity, environment and lead to the co-introduction of parasites and diseases (Trujillo-González et al., 2019; Saba et al., 2020a).

The educational level seems to be the most important factor influencing the attitude of the store owners. This follows the finding of Aminrad, Zakaria & Hadi (2011), who identified that age and educational level influenced environmental awareness. This is likely because those with a higher educational background are more likely to have the opportunity of being exposed to information regarding the need for conservation making them better informed compared to the less educated individuals (Clusa et al., 2018). In this study, the high proportion of respondents in the Bachelor’s category (>50%) who agreed that alien fishes should be allowed in Malaysia may be due to the low representation of this category in the sample. Furthermore, they may have been influenced by the fact that they gain economically from the sale of these fishes. This finding is also in tandem with Oxley, Waliczek & Williamson (2016), who found a significant association between educational level and membership of environmental organizations.

The significant association between the store owners’ attitudes concerning alien freshwater ornamental fish in Malaysia and the contribution of alien fishes to their income signifies that those who benefit more from alien fishes are in greater support of allowing alien fishes to be cultured and traded in Malaysia. This also gives an idea of the importance of alien fishes in their business (Ng, 2016). Besides, the significant association that existed between their attitude and the number of years in business indicates that those with more experience have likely gained a better understanding of the positive economic gains of the alien fishes. There was a significant relationship between attitude and educational level, and most of those who agreed that alien fishes should be allowed in Malaysia (79%) had secondary level education. This signifies the possibility that they probably are less knowledgeable of the existing or potential negative ecological impacts or believe that such impacts are not enough reason to disallow them considering the economic benefits they gain from them.

The significant associations between store owners’ monthly incomes versus fish sales as the main source of income and contribution of fish business to income level signify the importance of the fish component of their businesses. Moreover, alien ornamental fish species generally contribute to a good proportion of their fish income. Most of the store owners who answered “yes” to whether fish is the main source of income also agreed that alien fishes should be allowed in Malaysia and *vice versa*. This shows the importance of alien freshwater ornamental fish to the business of that section of the respondents. Besides, the negative relationship as observed between the numbers of available alien fishes versus years in business may be an indication that those who have stayed longer in the business are probably more aware or more concerned about the potential negative impacts of alien fishes.

## Conclusion

In conclusion, the ornamental freshwater fish industry within Klang Valley is dominated by alien fishes which contribute over half of the income (51–100%) to the businesses of store owners, while most (78%) of the store owners earned between RM 2001 and RM 5000 of income/month. Moreover, the alien ornamental fishes contribute the most to the income from the ornamental fish sale of about 42% of the store owners. Interestingly, we also found that educational level directly influences the environmental awareness toward alien ornamental fish culture and sale in Malaysia. While the mismanagement of alien ornamental fishes could give various negative ecological impacts, the socio-economic benefits of the alien ornamental fishes also cannot be denied. Although this study took place within Klang Valley, which is the most densely populated area within Malaysia and where the highest number of ornamental fish stores can be found, the results as interpreted in this study may be different for other parts of the country. Finally, considering their importance, future studies should also consider the economic contribution of alien fishes to the businesses of fish farmers and fisherfolks, and their attitude towards the importation, breeding culture, and sales of these fishes.

## Supplemental Information

10.7717/peerj.10643/supp-1 Supplemental Information 1 Raw data.

10.7717/peerj.10643/supp-2 Supplemental Information 2 Questionnaire in English and Bahasa Melayu.

## Additional Information and Declarations

### Data Availability

The following information was supplied regarding data availability: Questionnaire responses are available in the Supplemental Files.
