# Supplementary material for: Economic contribution and attitude towards alien freshwater ornamental fishes of pet store owners in Klang Valley, Malaysia
Source: PeerJ. 2021 Jan 13;9:e10643. doi: 10.7717/peerj.10643 (PMC7811282; doi:10.7717/peerj.10643)
Supplement: Supplemental Information 2 [file peerj-09-10643-s002.docx]

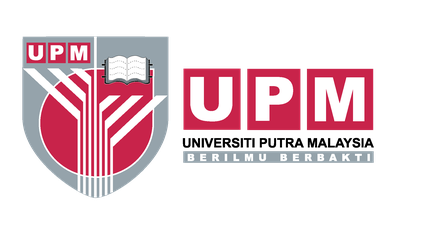


**English Version**

**QUESTIONNAIRE ON ECONOMIC IMPORTANCE ALIEN FISHES AND ATTITUDE OF PET STORE OWNERS**

**IN KLANG VALLEY, MALAYSIA**

**Section A: Demographic Profile**

1. Gender: Male Female
2. Age group: <18 18-25 26-35 36-45 46-55 >55 years old
3. Ethnicity: Malay Indian Chinese Others _____________________
4. Education: No education Primary Secondary BSc. MSc. PhD.

1. Monthly income: < RM2,000 RM2,000-5,000 RM5,001- RM10,000 > RM10,000
2. Marital status: Single Married Divorced

**Section B: Economic Importance of Alien Fishes**

1. Which of these do you own or manage? Fish farm Fish hatchery Fish pet shop None
2. How many years have you been in the business? 0-5 6-10 11-20 21-30 > 30 years
3. Do you sell other non-fish aquarium feed and accessories? Yes No
4. Is selling fish your main source of income? Yes No
5. If **NO** for Question 4, what is your main source of income? ____________________________
6. What is your other source of income? ____________________________________________
7. Which fish species do people buy most?

1. ________________________________ 2._____________________________ 3.________________________________ 4._____________________________

1. Your income level is mainly due to your engagement in fish business

Strongly disagree Disagree Agree Strongly agree

1. You also breed the fishes in your farm/shop Yes No

1. Alien fishes contribute the most to your fish related business income

Strongly disagree Disagree Agree Strongly agree

1. What percentage of your income comes from fish business? ______________________%
2. How many fish species do you have currently? _________________________________
3. How many fish approximately do you sell in a day? ___________________________
4. How much income approximately do you make from fish sale in a day? RM____________________

15. Alien fishes should be allowed to trade and culture in Malaysia for business purpose and economic generation

Strongly disagree Disagree Agree Strongly agree

**THANK YOU**

**THE END OF QUESTIONNAIRE**


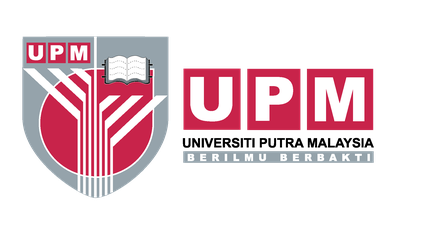


**Versi Bahasa Melayu**

**SOALAN BERKENAAN KEPENTINGAN EKONOMI IKAN PENDATANG DAN SIKAP PEMILIK KEDAI HAIWAN KESAYANGAN DI LEMBAH KLANG, MALAYSIA**

**Bahagian A: Profil Demografi**

1. Jantina: Lelaki Perempuan
2. Umur: <18 18-25 26-35 36-45 46-55 >55 tahun
3. Etnik: Melayu India Cina Lain-lain ______________________
4. Pendidikan: Tiada Rendah Menengah Ijazah Sarjana Falsafah

1. Pendapatan bulanan: < RM2,000 RM2,000-5,000 RM5,001- RM10,000 > RM10,000
2. Status perkahwinan: Bujang Berkahwin Bercerai

**Bahagian B: Kepentingan Ekonomi Ikan Pendatang**

1. Manakah antara yang anda miliki atau uruskan?

Ladang ikan Pusat penetasan ikan Kedai ikan hiasan Tiada

1. Sudah berapa tahun anda berniaga? 0-5 6-10 11-20 21-30 > 30 tahun

1. Adakah anda menjual selain makanan dan aksesori akuarium ikan? Ya Tidak
2. Adakah menjual ikan adalah pendapatan utama anda? Ya Tidak
3. Jika **TIDAK** untuk Soalan 4, apakah sumber pendapatan utama anda? ____________________________
4. Apakah sumber pendapatan anda yang lain? ____________________________________________
5. Apakah spesies ikan yang paling banyak dibeli?

1. ________________________________ 2._____________________________ 3.________________________________ 4._____________________________

1. Tahap pendapatan anda adalah disebabkan oleh penglibatan anda di dalam perniagaan ikan

Sangat tidak setuju Tidak setuju Setuju Sangat setuju

1. Anda juga membiakkan ikan di ladang/kedai anda Ya Tidak

1. Ikan pendatang penyumbang tertinggi untuk pendapatan perniagaan anda yang berkaitan dengan ikan

Sangat tidak setuju Tidak setuju Setuju Sangat setuju

1. Berapa peratus pendapatan anda datang dari perniagaan ikan? ______________________%
2. Berapa banyak spesies ikan yang anda miliki? _________________________________
3. Kira-kira berapa banyak ikan yang anda jual dalam sehari? ___________________________
4. Kira-kira berapa banyak pendapatan yang anda dapat dengan menjual ikan setiap hari? RM________________
5. Ikan pendatang harus dibenarkan berniaga dan dikultur di Malaysia untuk tujuan perniagaan dan penjanaan

ekonomi

Sangat tidak setuju Tidak setuju Setuju Sangat setuju

**TERIMA KASIH**

**SOALAN TAMAT**
